# Uso de tecnologías de información y comunicación para promover la autogestión de ulceras por pie diabético*

**DOI:** 10.15649/cuidarte.2254

**Published:** 2022-10-17

**Authors:** Ana Maria Murillo Salamanca, Alejandra María Alvarado-García

**Affiliations:** 1 Universidad pedagógica y tecnológica de Colombia UPTC. Tunja Colombia. Email: anmarmur@hotmail.com Universidad Pedagógica y Tecnológica de Colombia Universidad pedagógica y tecnológica de Colombia Tunja Colombia anmarmur@hotmail.com; 2 Universidad Antonio Nariño, Universidad de Antioquia. Medellín, Colombia. Email: alalvarado@uan.edu.co Universidad de Antioquia Universidad de Antioquia Medellín Colombia alalvarado@uan.edu.co

**Keywords:** Diabetes Mellitus, Pie Diabético, Promoción de la Salud, Intervención Educativa, Teoría de Enfermería, Tecnología de la Información, Diabetes Mellitus, Diabetic Foot, Health Promotion, Educational Intervention, Nursing Theory, Information Technology, Diabetes Mellitus, Pé Diabético, Promogao da Saúde, Intervengao Educativa, Teoria de Enfermagem, Tecnologia da Informagao

## Abstract

**Introducción:**

El uso de las tecnologías de la información y comunicación en las intervenciones educativas de enfermería facilita los procesos de autogestión para lograr la adaptación en las condiciones crónicas de salud.

**Objetivo:**

Evaluar el efecto de una intervención educativa de enfermería para promover la autogestión en la prevención de ulceras por pie diabético en adultos diabetes mellitus tipo 2 en Colombia mediante la inclusión de las tecnologías de la Información y comunicación.

**Materiales y Métodos:**

Estudio cuasi experimental con medición pre y post intervención realizado con 82 adultos que asistían a la consulta de enfermedades crónicas de un hospital de segundo nivel de atención en el departamento de Boyacá Colombia Los grupos fueron asignados aleatoriamente a los grupos experimental y comparación. Los datos fueron recolectados a partir de una ficha de caracterización y un cuestionario de autogestión.

**Resultados:**

no hubo diferencias significativas entre los dos grupos de experimental y comparación en términos de puntaje de autogestión, previo a la intervención. Sin embargo, el puntaje promedio de autogestión del grupo experimental fue significativamente mayor que el del grupo control 6 semanas después de la intervención (p<0.005) el resultado primario fueron los comportamientos de autogestión dados desde el cuidado de los pies. Se usaron las pruebas no paramétricas de Wilcoxon y Mann Whitney.

**Discusión:**

las intervenciones de enfermería deben soportarse en enfoques teóricos propios de la disciplina, que permita visualizar resultados específicos, en este caso la autogestión la cual requiere de estrategias como el conocimiento, la habilidad y el soporte social que apoyaran la adaptación para las situaciones de enfermedad crónica.

**Conclusiones:**

la intervención educativa a partir del uso de las tecnologías de la información y comunicación mejoro la autogestión para la prevención de heridas en pie diabético, logrando las personas un cambio en su comportamiento.

## Abstract (en)

**Introduction:**

Using information and communication technologies in nursing educational interventions facilitates self-management processes to adapt to chronic health conditions.

**Objective:**

To evaluate the effect of a nursing educational intervention to promote self-management and prevent diabetic foot ulcers in Colombian adults with type 2 diabetes mellitus through information and communication technologies.

**Materials and Methods:**

A quasi-experimental, pre- and post-intervention study was conducted with 82 adults attending the chronic patient consultation of a second-level hospital in the department of Boyacá, Colombia. Participants were randomly allocated to the experimental and comparison groups.

**Results:**

There were no significant differences between the experimental and the comparison groups in terms of preintervention self-management scores. However, the experimental group's mean self-management score was significantly higher than the control group's six weeks after the intervention (p<0.005); the primary outcome was self-management behaviors from foot care. Nonparametric Wilcoxon and Mann Whitney tests were used.

**Discussion:**

nursing interventions should be supported by theoretical approaches specific to the discipline, which allow visualizing specific results, in this case self-management, which requires strategies such as knowledge, skills and social support that will support adaptation to situations of chronic illness.

**Conclusions:**

The educational intervention based on the use of information and communication technologies improved self-management for preventing diabetic foot injuries, achieving a change in people's behavior.

## Resumo

**Introdujo:**

O uso de tecnologías de informado e comunicado nas intervengóes educativas de enfermagem facilita os processos de autogestao para alcangar a adaptado em condigóes crónicas de saúde.

**Objetivo:**

Avaliar o efeito de uma intervengao educativa de enfermagem para promover o autogerenciamento na prevengao de úlceras do pé diabético em adultos com diabetes mellitus tipo 2 na Colómbia por meio da inclusao de tecnologias de informagao e comunicagao.

**Materiais e Métodos:**

Estudo quase experimental com mensuragao pré e pós-intervengao realizado com 82 adultos atendidos no ambulatório de doengas crónicas de um hospital de atengao secundária do departamento de Boyacá, Colómbia. Os grupos foram aleatoriamente designados para o experimento e comparagao grupos. Os dados foram coletados a partir de uma ficha de caracterizagao e de um questionário de autogestao.

**Resultados:**

nao houve diferengas significativas entre os dois grupos experimental e de comparagao quanto ao escore de autogestao, antes da intervengao. No entanto, a pontuagao média de autogerenciamento do grupo experimental foi significativamente maior do que a do grupo controle 6 semanas após a intervengao (p<0,005), sendo o desfecho primário os comportamentos de autogestao dados a partir do cuidado com os pés. Foram utilizados os testes nao paramétricos de Wilcoxon e Mann Whitney.

**Discussao:**

As intervengóes de enfermagem devem ser apoiadas por abordagens teóricas específicas da disciplina, que permitam a visualizagao de resultados específicos, neste caso a autogestao que requer estratégias tais como conhecimentos, competencias e apoio social que apoiem a adaptagao a situagóes de doenga crónica.

**Conclusoes:**

A intervengao educativa baseada no uso de tecnologias de informagao e comunicagao melhorou a autogestao para a prevengao de lesóes no pé diabético, proporcionando as pessoas uma mudanga no seu comportamento.

## Introducción

La diabetes mellitus tipo 2(DM2) es una enfermedad crónica que se encuentra dentro de las principales causas de morbimortalidad en el mundo. Más de 425 millones de personas viven actualmente con diabetes^1^.Según la Organización Mundial de la Salud (OMS) es la cuarta causa de muerte a nivel global^2^.La Federación Internacional de Diabetes (FID) estima que más de 134.4 millones de adultos mayores viven con diabetes y se prevé que esta cifra aumente a 252.8 millones al 2035 lo que equivale a un 80% de la población actual^1^.

La tasa de complicaciones de la diabetes a nivel microvascular se estima en un 27,2% y a nivel microvascular en un 53,5%^3^^,^^4^. La neuropatía diabética es la complicación más frecuente y precoz de la diabetes. A pesar de ello suele ser la más tardía en diagnosticar. Su prevalencia es difícil de establecer debido a la ausencia de criterios diagnósticos unificados y las formas clínicas, es por tanto que su evolución y gravedad se correlacionan con la duración de la enfermedad, el mal control metabólico y la aparición de complicaciones^5^.

La OMS reporta que del 15 al 20% de la población diagnosticada con DM2 ha experimentado úlceras del pie diabético durante su vida y más de 1 millón de pacientes pierde su pie anualmente, lo que quiere decir que cada 30 segundos ocurre una amputación secundaria a la diabetes^6^. Este tipo de complicaciones generan un incremento en los recursos tanto para los sistemas sanitarios y familiares, por las hospitalizaciones prolongadas y por las discapacidades secundarias para el paciente y los cuidadores^7^.

La evolución de estas comorbilidades depende en gran medida de la ausencia de autocuidado a través de hábitos de vida protectores como el control metabólico, el ejercicio físico, el tratamiento farmacológico y la autogestión de la enfermedad comprendida como la responsabilidad de la persona para hacerse participe de su cuidado a partir de conocimientos, habilidades y confianza en la participación de su cuidado^8^.

Es necesario resaltar que la población a la cual se le atribuye con mayor frecuencia este tipo de comorbilidades es la población adulta mayor, la cual, debido a factores asociados al proceso de envejecimiento como su estado de salud, aspectos cognitivos, psicosociales, ausencia de redes de apoyo^9^ e incluso creencias sobre su enfermedad pueden generar complicaciones, necesitando de un mayor apoyo para lograr un control de su diabetes.

Actualmente los programas para la prevención y tratamiento de la diabetes en los últimos años se han centrado en generar empoderamiento por parte del paciente a partir del desarrollo de intervenciones que les permita identificar sus propios problemas y gestionar su enfermedad^10^. En este orden de ideas, varias teorías han intentado explicar este fenómeno; es así como la teoría de adaptación a las enfermedades crónicas de salud^11^ afirma que “los procesos adaptativos en condiciones crónicas de salud incluyen el empoderamiento, la adopción de cambios, el soporte social, para lograr que el individuo tome responsabilidad para asumir el automanejo de su propia salud.” Vincular este supuesto a los programas puede lograr que el paciente con diabetes se adapte a la enfermedad, cambie su curso y genere la responsabilidad para alcanzar su cuidado^10^.

El proceso adaptativo en la enfermedad crónica incorpora la toma de responsabilidad del individuo sobre su condición, el cual es seguido por una búsqueda y procesamiento de la información para crear una conciencia de la necesidad de adaptación, reflejada en el sentido de un compromiso hacia su salud, para luego lograr un nivel de competencia que le permita alcanzar la habilidad. Es allí donde las intervenciones de cuidado deben reforzar las habilidades que tiene el individuo y proveer un soporte dado desde el acompañamiento que le permita alcanzar una normalidad desde lo esperado para su condición de salud^11^.

Una revisión sistemática reportó efectos positivos en los programas de educación, donde a partir de ellos se logra que el paciente incluya el cuidado de sus pies, los cuales se enfocan en brindar conocimiento sobre las mejores técnicas, entre estas el autocuidado de los pies como una práctica cotidiana, donde se incluyan hábitos como la inspección diaria, el lavado diario, el secado completo, el uso de zapatos adecuados, el corte de las uñas y el no manipular lesiones como callos en los pies., asumiendo el control regular y asistencia a sus consultas médicas^12^.

Algunos de estos programas han sido mediados por componentes interactivos basados en el uso de las tecnologías donde el paciente tiene la posibilidad de interactuar con estas herramientas y a su vez recibir información, y sentirse seguro frente a su uso^12^.

El uso de las tecnologías de la información y comunicación (TIC's) permite acceder de manera fácil y económica a información relacionada con la promoción y la gestión de la salud; cada vez se reconoce que su uso resulta ser costo efectivo para los sistemas de salud, ya que pueden alcanzar a numerosas poblaciones. Las TIC's combinadas con herramientas de monitoreo pueden mejorar variables como la actividad física, el peso, entre otras que resultan en beneficio para las personas con enfermedades crónicas^13^^,^^14^.

Por lo tanto, considerando el hecho de que la diabetes es altamente controlable a partir del empoderamiento y autogestión de la enfermedad, además la poca disponibilidad de estudios basados específicamente en intervenciones de enfermería para apoyar autogestión en la prevención por úlceras por pie diabético, mediados por tecnologías de la información y la comunicación, se espera que este trabajo contribuya al desarrollo de intervenciones validadas soportadas en componentes teóricos que den respuesta a una necesidad real. Por lo anteriormente expuesto se planteó como objetivo evaluar el efecto de una intervención educativa de enfermería para promover la autogestión en la prevención de ulceras por pie diabético en adultos diabetes mellitus tipo 2 en Colombia mediadas por las tecnologías de la Información y comunicación.

## Materiales y Métodos

Estudio cuasi experimental con grupo experimental y comparación, pre- test y pos-test. El grupo experimental recibió la intervención educativa y el grupo comparación recibió la intervención convencional. Se utilizó un muestreo no probabilístico por conveniencia, ya que se trabajó con grupos intactos, donde la asignación para cada grupo (experimental y comparación), fue aleatoria simple a partir de la consulta de enfermedades crónicas. La asignación a los grupos se realizó bajo la función de números aleatorios de Excel, y el software de cálculo utilizado fue el programa SPSS versión 20, la matriz de datos está disponible en Mendeley Data^15^.

Población y muestra: La población estuvo conformada por los adultos que asistían a la consulta de enfermedades crónicas de un hospital de segundo nivel de atención en el departamento de Boyacá Colombia, durante los meses de agosto de 2017 a febrero de 2018. El tamaño de la muestra para esta investigación se calculó mediante la siguiente expresión:




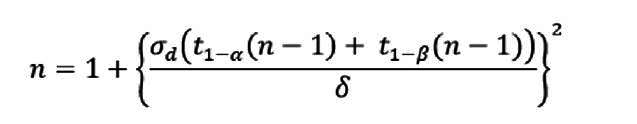




Por medio de la expresión anterior adoptando el mismo riesgo para el error tipo I y para el error tipo II, cuantificado en alfa= beta= 0,015. Por otra parte, se asumió como valor de A el 80% del valor de la desviación estándar, esto es, A= 0,8o. De esta manera al considerar los dos grupos que la investigación propone en su parte metodológica, k=2, el tamaño de la muestra requerido es de 41 pacientes en cada uno de los grupos^16^.

Como criterios de inclusión se establecieron personas mayores de 18 años diagnosticadas con diabetes mellitus tipo 2, que supieran leer, tener una valoración que certifique que la piel esté íntegra, también que tuviesen equipo de telefonía móvil con funciones básicas, dotado para recibir mensajes de texto, y como criterios de exclusión la presencia de lesiones en piel.

En este estudio como variables independientes se definieron la intervención educativa de enfermería mediada por TIC's. y la variable dependiente el nivel de autogestión para la prevención de úlceras por pie diabético.

En ausencia de instrumentos reportados por la literatura que permitieran valorar el nivel de autogestión; se construye un formato Ad Hoc que permitió medir la autogestión bajo los conceptos de la teoría de adaptación en condiciones crónicas^11^ a partir de la taxonomía Nanda^14^.

Los indicadores NOC permiten identificar, nombrar y medir los resultados de la práctica de enfermería. Se seleccionaron 6 indicadores NOC acordes con elementos de la autogestión, como son: conocimiento, experiencia y soporte para la prevención de úlceras por pie diabético.

**Figura 1:**
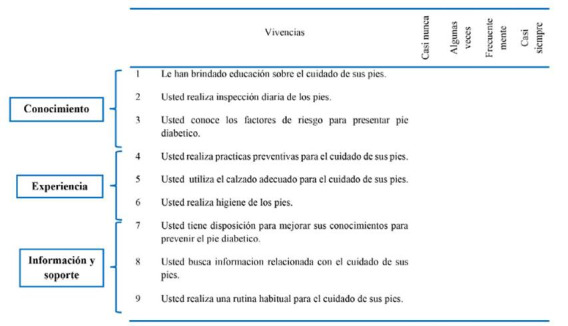
Instumento Adhoc- Boyaca -Colombia- 2017-2018

Se realizó el formato Ad Hoc el cual requirió hacer la primera validación de contenido, donde participaron 3 enfermeras expertas en la temática, 2 con maestría 1 con doctorado quienes trabajan con pacientes diabéticos y con heridas. Ellas realizaron la validez de contenido. Después de su revisión y retroalimentación se obtuvo el formato Ad hoc con un total de 9 ítems cuantificados por una escala tipo Likert de 1 a 4 donde 1 equivale a casi nunca, 2 algunas veces 3 frecuentemente 4 casi siempre para las tres dimensiones: conocimientos (3 ítems), experiencia (3 ítems), información y soporte (3 ítems) donde el valor mínimo obtenido de nivel de autogestión es 9 y el valor máximo 36. (Ver Figura 1).

Puntuación fue definida: **Casi nunca:** la situación no ocurre o ha pasado 1 vez; **Algunas veces:** la situación se ha presentado más de 1 vez. **Frecuentemente:** la situación ocurre más de 2 veces. **Casi siempre:** la situación ocurre 4 veces o más.

**Figura 2:**
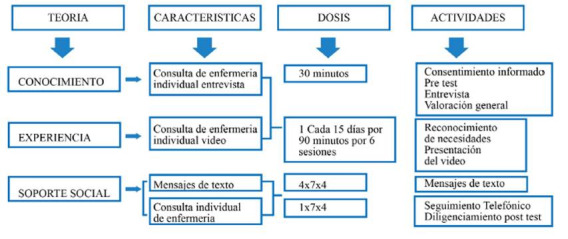
Relación de la teoría de Mediano Rango de Adaptación a las Condiciones de Salud Crónicas de Bruckner y Hayden con la investigación

El marco teórico utilizado para fue el propuesto por Buckner EB, Hayden SJ.,^11^ el cual hace referencia a los procesos de adaptación a las enfermedades crónicas. Este incluye componentes como el conocimiento, la experiencia y el soporte social como determinantes para alcanzar la autogestión (Ver Figura 2). Del mismo modo la inclusión de esta teoría de mediano rango derivado del modelo de adaptación de Callista Roy ofrece elementos para mejorar la práctica en enfermería ya que permite visibilizar intervenciones más humanas e integradoras; donde la persona se hace partícipe de su cuidado adhiriéndose al tratamiento de forma natural.

**Grafica 1:**
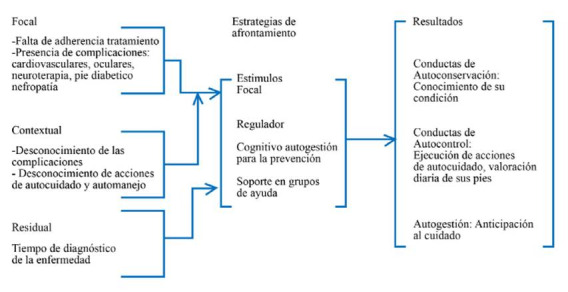
Protocolo de la intervención

### Descripción de la intervención:

*Primera Fase.* Denominada conductas de Auto conservación: Valoración y exploración de necesidades y significados de las personas. se realizó la entrevista a cada paciente, se diligenció el pre test por medio del Instrumento Ad hoc.

*Segunda Fase.* Denominada conductas de autocontrol: en esta fase se presentó cada paciente un video educativo, el cual se realizó a partir de lo reportado en la evidencia científica sobre la educación a un paciente diabético y así mismo los contenidos por expertos en el área de medicina interna.

### Los temas centrales del video fueron:

1. Enfermedad: diabetes mellitus tipo. Que es la diabetes mellitus tipo, Signos y síntomas.
2. Complicaciones de la enfermedad. Como vivir con diabetes: Componentes del cuidado: tratamiento, alimentación, ejercicio, conductas de autogestión.
3. Cuidado de los pies: Inspección diaria de los pies, Higiene de los pies: baño; temperatura acorde, secado, corte de las uñas Hidratación de la piel. La importancia del uso de medias diarias. Calzado adecuado. Identificación de signos de alarma. Como hacer una rutia diaria del cuidado de los pies.

Durante esta fase se realizó una reafirmación de las diferentes acciones de cuidado planteadas, Al final de esta sesión se entregó una copia del video presentado con el fin de que sea un elemento de motivación y de recordación en casa para que pudieran reproducirlo las veces que ellos consideraran pertinente.

*Tercera fase.* Denominada autogestión anticipación al cuidado promover prácticas centradas en generar acciones de empoderamiento y frente a la enfermedad: se diseñaron 30 mensajes de texto acordes con los elementos de la autogestión: conocimiento, experiencia y soporte; estos mensajes tenían como objetivo evocar diferentes recordatorios para reforzar y generar conductas para el cuidado de sus pies. Los mensajes se enviaron 1 vez durante tres días a la semana por 4 semanas, los días lunes, miércoles y viernes y el día sábado por medio de una llamada telefónica se hacia el seguimiento y con ellos se confirmaba el recibido de los mismos. Luego de las 4ta semana de realizada la intervención se captaron nuevamente los participantes y se les realizo el pos test, en este orden de ideas la intervención tuvo una duración de 6 semanas desde su inicio hasta la finalización.

**Figura 3:**
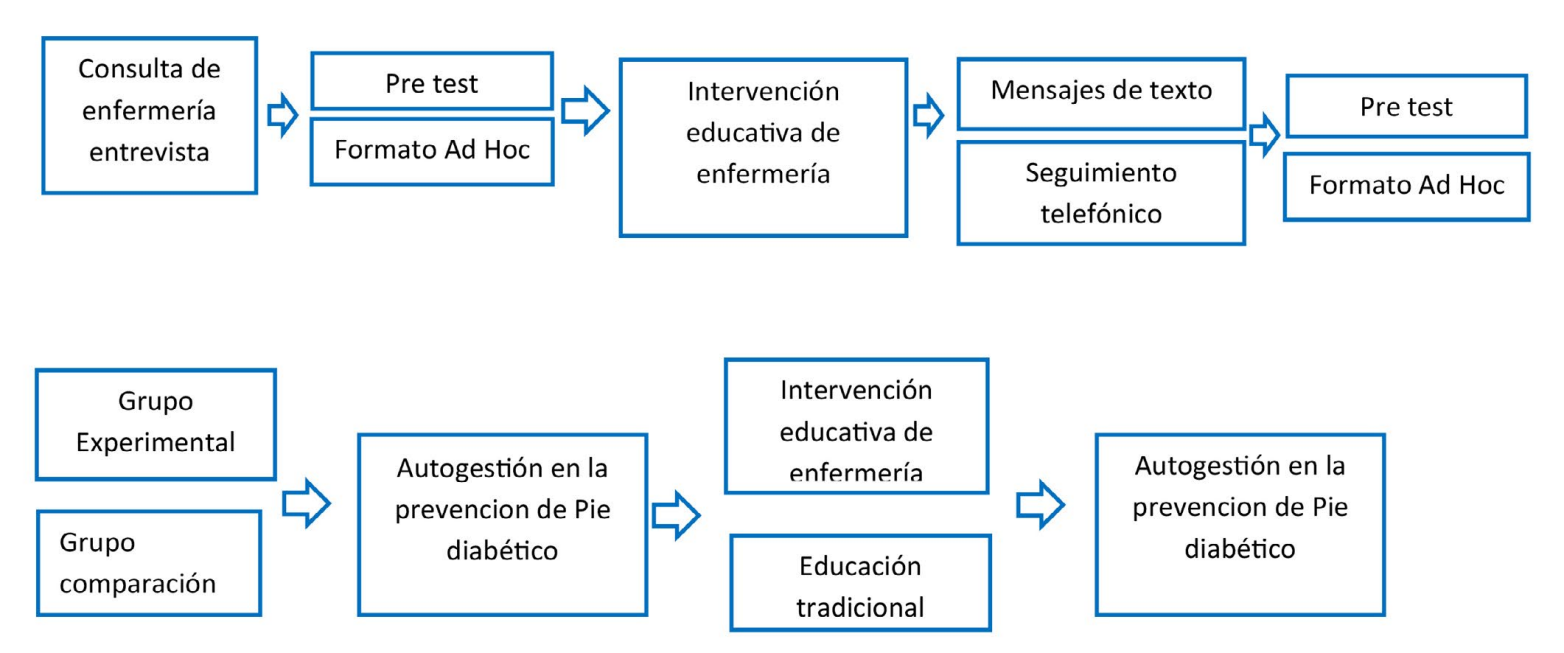
Diagrama del estudio

Respecto al análisis de los datos de las variables sociodemográficas, se utilizó una tabla de frecuencia y estadística descriptiva que permitió contextualizar la información de los participantes tanto del grupo experimental como del grupo comparación. Se determinó el nivel de significancia, se emplearon las pruebas no paramétricas Wiconxon y la prueba por resultados de U de Mann Whitney con el fin de comparar los puntajes y la diferencia entre grupos. La base de datos fue almacenada en Mendeley Data^15^.

La investigación se acogió a los lineamientos de la resolución 008430 de 1993 del Ministerio de Salud de la Republica de Colombia, la cual clasifica la investigación con un riesgo mínimo, se contó con el aval de la Subcomisión de investigación de la Acta No 006.2017 y los avales de la institución donde se llevó a cabo la investigación, se aplicó el consentimiento informado y se respetaron los principios de autonomía, confidencialidad de los participantes.

## Resultados

Este estudio garantizo el total de la muestra, 82 personas distribuidas en dos grupos: experimental y comparación. En cuanto a las características sociodemográficas, en el grupo experimental se encontró que las mujeres representaron el 80% y los hombres un 20% de la población mientras que en el grupo comparación se obtuvo un porcentaje de mujeres del 68.(05) y de los hombres el 32.(04) evidencia una mayor participación del género femenino asociado al aumento en la prevalencia de la enfermedad en Colombia donde para el año 2013 la diabetes tenía una prevalencia de 6.(70)en hombres y 6.(10) en mujeres; para el año 2016 se observa una variación puesto que mostro una prevalencia de 8.(50) en mujeres y hombres en 7.(60) Ver Tabla 2.

**Tabla 2:** Datos sociodemográficos de la población

Variables	Grupo Experimental G1 N: 41	Grupo Comparación G2 N:41	Valor P
Genero			0,312
Femenino	33(80.5)	28(68.3)	
Masculino	8(19.5)	13(31.7)	
Estado civil			0,229
Soltero	12(29.3)	9(22,0)	
Casado	2(4,9)	4(9.8)	
Divorciado	20(49.8)	20(48.8)	
Viudo	7(17.1)	4(9.8)	
Unión libre	0(0,0)	4(9,8)	
Nivel educativo			0,164
Primaria	7(65,9)	30(73,2)	
Bachillerato	9(22,0)	11(26,8)	
Técnico	1(2,4)	0(0,0)	
Universitario	4(9,8)	0(0,0)	
Tiempo de diagnóstico de la enfermedad			0,367
Menor a 1 año	9(22,0)	4(9,8)	
Entre 3 a 5 años	18(43,8)	21(51,2)	
Mayor a 10 años	14(34,1)	16(39,0)	
Tiempo de tratamiento de la enfermedad			0,282
Menor a 1 año	11(26,8)	5(12,2)	
Variables	Grupo Experimental G1 N: 41	Grupo Comparación G2 N:41	Valor P
Entre 3 a 5 años	17(41,5)	21(51,2)	
Mayor de 10 años	13(31,7)	15(36,6)	
Conocimientos previos de la enfermedad			0,100
Si	21(51,2)	20(48,8)	
No	20(48,8)	21(51,2)	
Apoyo familiar			0,100
Si	26(63,4)	27(65,9)	
No	15(36,6)	14(34,1)	

Prueba estadística utilizada: chi-cuadrado de homogeneidad

Las necesidades encontradas durante la primera parte de la intervención de los 2 grupos, evidenciaron desconocimiento de la enfermedad, vacíos en cuanto al control de la glicemia y la ausencia de rutinas en el cuidado de los pies. Este análisis permitió construir el material para el video y los mensajes de texto. Luego se hace énfasis en lo que cada persona necesitaba conocer, mantener y/o reforzar en cuanto a conocimiento y habilidad.

Para determinar el nivel de autogestión se tuvo previsto un rango mínimo 9 y el valor máximo 36, al valorar cada elemento: conocimiento, experiencia y soporte presentan un valor mínimo de 3 y máximo de 12, cuantificado de acuerdo al número de ítems del documento Ad Hoc y la escala Likert.

**Figura 4:**
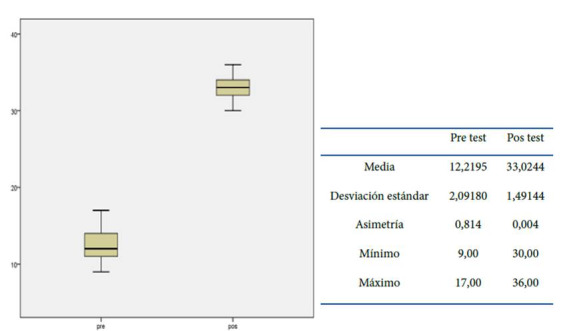
El nivel de autogestión de los pacientes, en el momento del pre- test y pos test del grupo experimental

El nivel de autogestión de los pacientes, en el momento del pre- test del grupo experimental obtuvo una mediana de 12,2, mientras que el grupo comparación fue de 11,41, y en el post test, la mediana fue de 33,02 y 11,29 respectivamente, mostrando un valor p de <0.001, Ver Figura 4.

**Figura 5:**
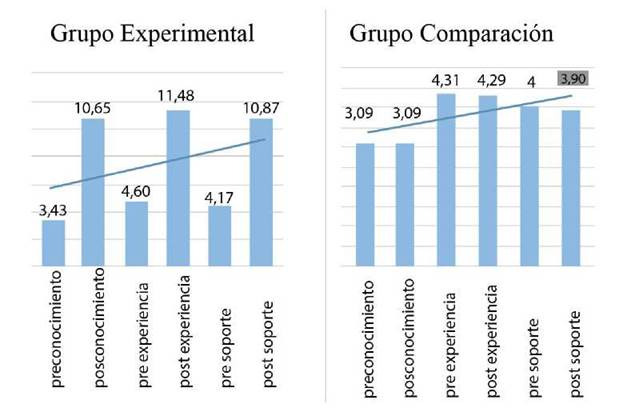
Nivel de autogestión previo y posterior del grupo experimental y comparación -Boyacá-Colombia- 2017-2018

Los elementos de la autogestión en el grupo experimental que tuvieron una mejora significativa fue en primer lugar el conocimiento, seguido por la experiencia y finaliza con el soporte, como se puede apreciar en la Figura 5.

Respecto a la prueba U de Mann Whitney En el pre test, no se encontró diferencia Estadísticamente significativa entre los dos grupos (valor-p = 144). Por su parte en el pos test se encontró diferencia Estadísticamente significativa entre los dos grupos (valor-p < 0,01).

## Discusión

El estudio reporto cambios significativos en los comportamientos de los adultos con DM2 frente a la prevención de ulceras por pie diabético, después de realizar una intervención educativa de enfermería para promover la autogestión a partir de elementos como conocimiento, experiencia y soporte social, validando la proposición teórica propuesta por Bucker y col^11^ la cual sustenta que a partir de una intervención de enfermería se logra conocer las necesidades en salud de las personas teniendo en cuenta su experiencia frente a su enfermedad para educar sobre sus propios requerimientos de cuidado.

Por otro lado, cuando se generan acciones para promover el soporte social enmarcadas dentro de un seguimiento y comunicación continua, los procesos adaptativos en personas con condiciones crónicas de salud mejoran promoviendo seguridad en la toma de su propia responsabilidad para el automanejo de su salud.

La puntuación para el nivel de autogestión del grupo experimental después de la intervención mejoro luego de 6 semanas de seguimiento, mientras que en el grupo comparación no se presentaron cambios entre el inicio y la finalización^17^^,^^18^.

El tiempo que duró la intervención conocida como la dosis, fue dada en 6 semanas sin tener en cuenta el primer encuentro donde se realizaron la fase 1 y 2, tiempo que es congruente con lo reportado en la literatura^19^ donde autores como de Menino, et^18^ al y Silva et^20^ sostienen que el promedio de los programas educativos oscila entre 2,75 a 4 sesiones para los pacientes diabéticos y tener una respuesta positiva de la intervención^21^.

Es necesario comprender que las personas con enfermedades crónicas en este caso DM2 no solo se trata a través del control de signos se han identificado aspectos propios del paciente que van desde el reconocimiento de la enfermedad hasta la vivencia de los síntomas lo que hace que la persona perciba necesario el involucrar rutinas de cuidado diario ya que se inician a ejercer cambios en el cuerpo cuando se realizan de manera constante y cuando no se continúan aparecen novedades que desencadenan en ellos signos de alerta. En la medida en que las personas consiguen la autogestión son más emprendedoras en la búsqueda y procesamiento de la información ya que es un soporte que le permite anticiparse al cuidado y a la adaptación de la enfermedad^21^.

La participación de los pacientes en aspectos relacionados con su propia salud refleja como intervenciones educativas con énfasis en prevención logran empoderar a los pacientes, reducir efectos secundarios y complicaciones en el cuidado de sus pies, en ocasiones la educación convencional brinda información sobre la enfermedad que no se contextualiza en las necesidades personales y no trasciende a que la persona reconozca como debe cuidarse porque solo revela que es la patología.

Autores como Bodenheimer, Lorig, Holman y Grumbach^22^ han denotado diferencias entre la educacion tradicional y/o convencional y la educacion basada en la autogestion. La educación convencional pretende que al paciente se le faciliten una serie de objetivos, los cuales debe cumplir en un tiempo determinado, conocido esto como una meta en salud, por otro lado, el objetivo de la autogestión es incrementar estrategias que le permitan mejorar los resultados clínicos y por ende sentirse que es capaz de desarrollar por sí solo, en otras palabras, alcanzar una autoeficacia. En este orden de ideas la autogestión se alcanza a partir de enseñar habilidades a las personas con el fin de utilizar herramientas internas y de su contexto para hacerse cargo de su enfermedad, lo que se entiende como aprender a dar respuesta a sus necesidades, utilizar los recursos con los que cuenta y permitirse la participación con un equipo interdisciplinario para poder llegar a un resultado positivo en beneficio de su condición^23^.

La intervención mejoró el nivel de conocimiento de los adultos mayores en un intervalo corto, pudiéndose atribuir al soporte brindado a partir de estrategias como el uso de las TIC's, logrando afirmar como éstas abren posibilidades tanto para los pacientes y sus cuidadores familiares facilitando procesos de adaptación frente a la situación de salud^23^^-^^24^.

La implementación de las TIC's en diferentes poblaciones quienes padecen de enfermedades crónicas no transmisibles y a su vez se encuentren en condiciones de vulnerabilidad económica y social se convierten en un reto para los países en vía de desarrollo es por este motivo que la generación de conocimiento en esta área lograra impactar en las políticas en beneficio de las poblaciones y hacer evidente el rol de la enfermería^25^.

Las TIC's fueron recibidas favorablemente durante el desarrollo del estudio; la tecnología de la información en este caso el video educativo fue una ayuda para que las personas fortalecieran sus conocimientos y resolvieran dudas concernientes a la DMt2, la posibilidad de tenerlo en casa como un recordatorio les permitió a los adultos tener un interés por aprender a cómo cuidarse^26^.

Por otro lado, estudios como Houston T. et al^27^ afirman que los grupos de apoyo con TIC's ofrecen alternativas de ayuda a los pacientes ya que aumentan sus conocimientos y habilidades de autoayuda, tendiendo un estilo de afrontamiento efectivo.

La tecnología de la comunicación utilizada fue los mensajes de texto que se convirtieron en un método sencillo de seguimiento y apoyo; ya que muchas de las personas respondían los mensajes y confirmaban el recibido y la importancia de la información, a su vez la realización de la llamada telefónica 1 vez por semana permitió un acompañamiento en el proceso de la adquisición de la autogestión, en el grupo experimental^28^^-^^29^.

Baji Z, Zamani Alavijeh F, Shakerinejad G, Tehrani M.^29^ compararon el impacto de enviar mensajes de texto a través del SMS móvil con contenido en las ventajas y desventajas de autocuidado del pie en mujeres con diabetes tipo 2, y encontraron como después de la intervención el grupo experimental en comparación con el control se encontraron cambios significativos, soportando lo encontrado en el estudio.

Limitaciones: Se presentaron dificultades administrativas en los tiempos para obtener el aval en instituciones de salud para el desarrollo de la investigación educativa de enfermería. No se pudieron determinar los resultados de la intervención a largo plazo, por el tiempo que duró la investigación.

## Conclusión

Esta investigación permitió explorar el efecto de una intervención educativa de enfermería mediada con TIC's con la educación convencional, evidenciando que la intervención educativa genera un nuevo conocimiento sobre la autogestión; a la vez que potencia las capacidades de las personas para emprender acciones de cuidado respecto a la prevención de úlceras por pie diabético.

La utilización de tecnologías de salud móvil brinda un plus de ayuda que permite transferir la información y brindar un acompañamiento durante el proceso de formación de la autogestión, permitiendo el desarrollo de nuevos elementos de alcance para orientar la prevención y educación de los profesionales de enfermería

Del mismo modo este resultado deja entrever que la práctica de enfermería va más allá del cumplimiento de acciones rutinarias y asistenciales, ya que requiere de recursos intelectuales, para tomar decisiones y realizar acciones pensadas y reflexionadas, que respondan a las necesidades individuales de la persona basados en esquemas conceptuales propios.
